# Massive parallel sequencing in forensics: advantages, issues, technicalities, and prospects

**DOI:** 10.1007/s00414-020-02294-0

**Published:** 2020-05-25

**Authors:** David Ballard, Jakub Winkler-Galicki, Joanna Wesoły

**Affiliations:** 1 King’s Forensic Genetics, Faculty of Life Sciences and Medicine, King’s College London, 150 Stamford Street, London, UK; 2 Laboratory of High Throughput Technologies, Faculty of Biology, Adam Mickiewicz, University Poznan, 6 Uniwersytetu Poznanskiego Street, Poznan, Poland

**Keywords:** Nucleic acids, NGS, MPS, Sequencing by synthesis, Nanopore sequencing

## Abstract

In the last decade, next-generation sequencing (NGS) technology, alternatively massive parallel sequencing (MPS), was applied to all fields of biological research. Its introduction to the field of forensics was slower, mainly due to lack of accredited sequencers, kits, and relatively higher sequencing error rates as compared with standardized Sanger sequencing. Currently, a majority of the problematic issues have been solved, which is proven by the body of reports in the literature. Here, we discuss the utility of NGS sequencing in forensics, emphasizing the advantages, issues, the technical aspects of the experiments, commercial solutions, and the potentially interesting applications of MPS.

## Introduction

**Fig. 1:**
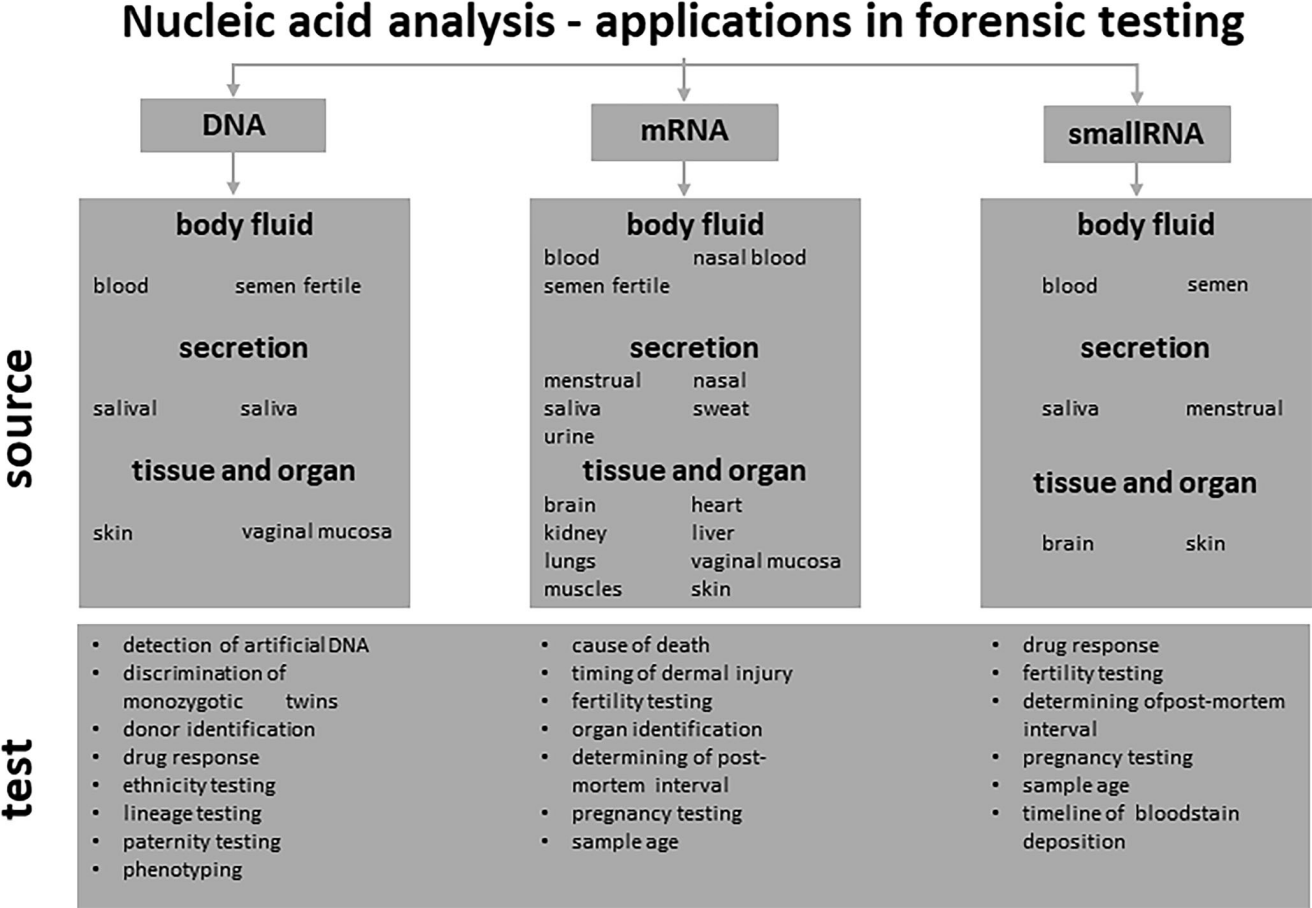
Most prevalent applications of nucleic acid analysis in forensic testing

Recent developments in sequencing technologies that have been introduced to research, diagnostic, and forensic laboratories have significantly improved the quality of nucleic acid analysis and increased the applicability of such analyses [1, 2]. Next-generation sequencing (NGS) methods effectively allow all types of nucleic acids to be sequenced, using a whole genome or targeted approach, with DNA, mRNA, and small RNA sequencing as standard analyses. In addition, larger-scale sequencing of specific RNA subtypes, such as long non-coding RNAs and snoRNA, as well as methylated DNA, have become possible with the introduction of NGS. The prospect of simultaneously analyzing a large number of markers such as STRs and SNPs in parallel with targeted mRNA and small RNA analysis makes MPS a very powerful, relatively easily applicable, tool in forensic laboratories (see Fig. 1).

### NGS methodology

Novel sequencing methods are characterized by a number of different technologies including sequencing by synthesis (SBS) following clonal nucleic acid amplification [3], nanopore sequencing [4], and single molecule sequencing in real time [5]. Although error rates of MPS platforms are higher in comparison with Sanger sequencing and vary between 0.5 and 15% depending on the platform used, this shortcoming can be overcome with a proper experimental design, namely a suitable sample coverage [2].

**Fig. 2:**
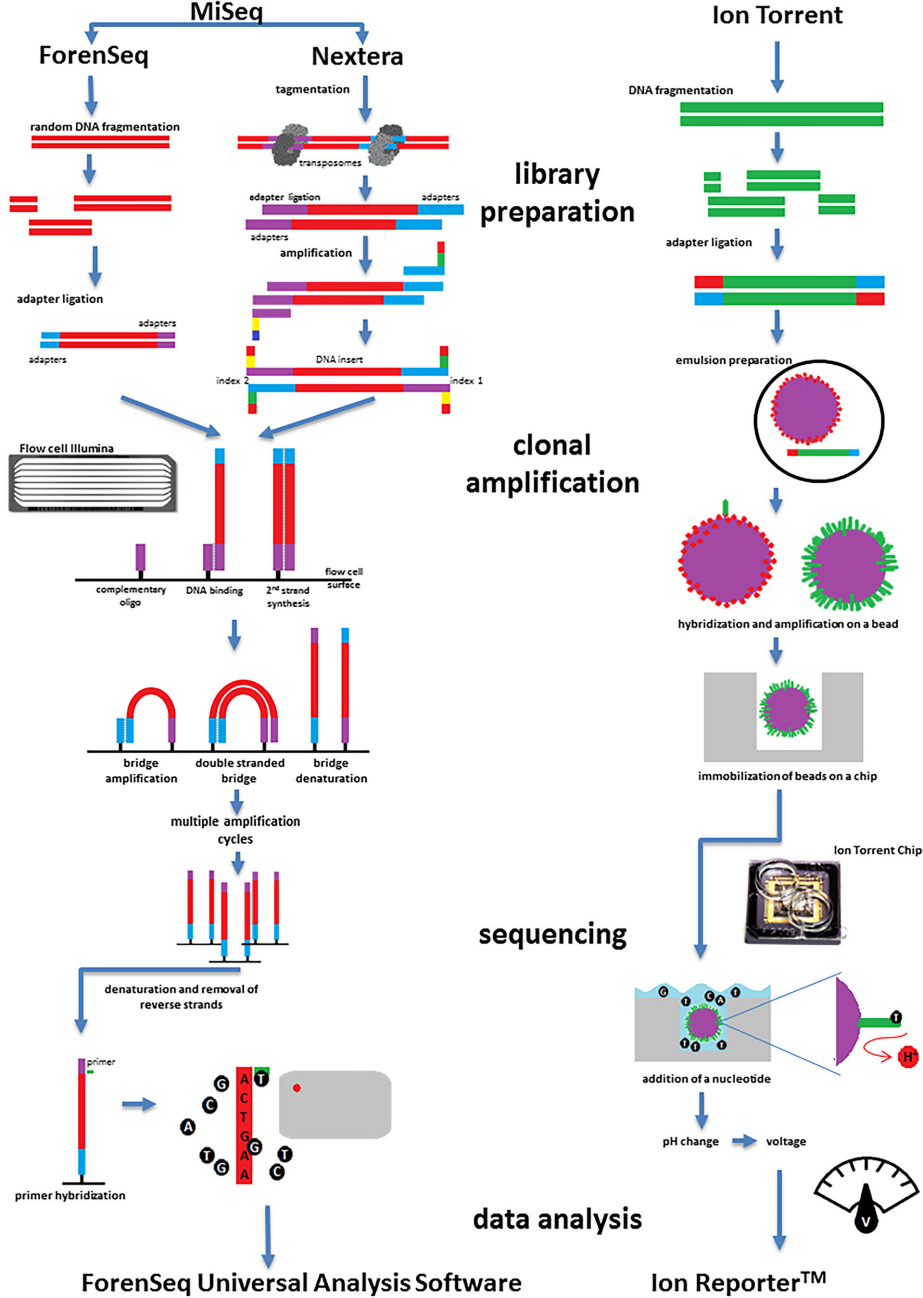
Schematic illustration of sequencing with MiSeq FGx and Ion Torrent, **a** MiSeq: Forenseq is a library prep used for STR and SNP sequencing (autosomal STRs, sex, geographical ancestry, and phenotypic SNPs); alternatively, Nextera is utilized for mtDNA sequencing. In the process of sample preparation, adaptors are added to DNA fragments in a two-step PCR reaction in order to enable DNA binding to a glass slide. In the next step, the fragments are clonally amplified on the slide and sequenced. The template strand is extended with one nucleotide at a time. The reaction of polymerization is halted due to the use of 3′-*O*-azidomethyl-dNTPs that are fluorescently labeled. The base incorporation is followed by removal of unincorporated bases and imaging using CCD camera. Subsequently, the 3′ block and the fluorescent tag on the incorporated nucleotide are removed and the reaction proceeds to the next cycle. **b** Ion Torrent: sample preparation of DNA fragments for sequencing on Ion Torrent is similar to the workflow utilized by Roche 454 sequencer, followed by amplification of adaptor-ligated DNA hybridized to beads using emulsion PCR (Margulies et al. 2005) [1]. The beads are distributed to microwells, where sequencing by synthesis occurs. The sensor located at the bottom of the well converts the changes in pH into a voltage signal proportional to the number of incorporated bases

Currently, there are but a few NGS bench-top sequencers that dominate the forensic genetics landscape: Illumina’s MiSeq FGx, ThermoFisher’s Ion Torrent PGM, and Ion S5. While Illumina implements cycle-based sequencing technology coupled with a reversible termination strategy of fluorescently labeled modified dNTPs, conceptually similar to Sanger sequencing [6], the Ion Torrent is a semi-conductor sequencer that measures pH changes—a consequence of the release of hydrogen ions during synthesis of DNA (see Fig. 2) [7]. While base substitutions are the most common sequencing errors generated during sequencing on Illumina machines, insertions and deletions are the most frequent errors introduced by the Ion Torrent PGM [4]. In the case of the latter, homopolymer stretches longer than 6 bp are the most difficult to call, since the correlation between the incorporated nucleotides and the change in detected voltage is not exactly to scale [4, 6]. Although the error rates generated by the Ion Torrent are higher (≥ 1%) [8] and DNA library preparation protocols can be more time consuming and cumbersome in comparison with the MiSeq workflow, the lack of optical scanning and cycle-based sequencing significantly reduces the time of DNA sequencing [9]. One notable advantage of the Ion Torrent platforms is the availability of automated library preparation and chip loading stations, which simplifies the workflow considerably. Recently, similar automation and liquid handling solutions have been proposed for many MiSeq workflows [10].

### NGS as a tool for STR and SNP genotyping

Traditional STR analysis with gel- or capillary-based electrophoresis (CE) estimates the repeat number of the STR marker through the size of the PCR amplicon. Analysis instead with NGS allows the full sequence of the PCR product to be determined including the STR repeat region and the surrounding flanking areas. Numerous studies have demonstrated that sequence-based STR analysis, as opposed to amplicon size-based analysis, results in a pronounced increase in allele variability for many forensically relevant STRs [11–15]. Even loci that appear to show no additional sequence variation in individual populations can be informative in others, as demonstrated by a study of four major population groups in the USA where only TPOX of the commonly used autosomal loci failed to show added sequence variation in any of the investigated populations [14]. Information regarding forensic STR reference sequences and the sites of known variation is available from the STRidER Website [16], while the STRSeq project is cataloging all observed sequence alleles for major forensic STR loci and this is searchable on NCBI [17].

Capturing the additional sequence variation that can be present in STR repeat and flanking regions can have many benefits above and beyond merely an improvement in discrimination power when using these markers for direct matching or relationship calculations. Previous work on STR allelic stutter proportions has proposed that stutter is associated with the longest uninterrupted repeat in the STR allele [18], and Van der Gaag et al. [19] have elegantly demonstrated with MPS data that the stutter ratio for an allele at a given locus is dependent on the specific sequence of that allele (i.e., the length of the longest uninterrupted repeat stretch, which can vary with alleles of the same length containing compound or complex repeat motifs). This introduces an additional MPS advantage compared with CE when analyzing STRs, namely that the increased information gained from the allele sequence can be used to predict stutter behaviors with more accuracy.

For DNA mixtures, the improvement in marker discrimination will by itself be highly beneficial for identifying extra sequence-specific alleles that would have previously been masked due to identical CE amplicon lengths, while the increased discrimination will also make it less likely for an individual to have an adventitious match between their profile and the alleles present within a mixed stain. The additional use of STR flanking variants, which can be detected with NGS methods, to aid mixture analysis has previously been highlighted through work carried out on deletion/insertion polymorphisms present in the flanking regions of STRs (DIP-STRs) [20]. Further, NGS studies on DNA mixtures have confirmed that mixture proportions are accurately reflected by read number [13].

One notable advantage of analysis by MPS rather than CE is that separation by amplicon size is no longer a requirement for multiplex STR assay design; this means that all STRs can be amplified using the smallest feasible amplicon length, improving amplification in cases of degraded DNA, and that the number of markers amplified simultaneously is no longer constrained by the fluorescent dye detection capabilities of electrophoretic systems hence heralding the possibility of co-amplifying increasing numbers of STRs at the same time. One use of this would be to allow co-amplification of large panels of Y and autosomal STRs, providing increased power for male/female mixed stain analysis, e.g., following sexual assault.

Amplification of forensic DNA samples by MPS is not limited to STR loci though, and SNP markers can also be analyzed either in combination with STRs or on their own. The advantages of analyzing SNPs for forensic samples with MPS rather than alternative technologies such as SNaPshot or SNP array systems, rest predominantly in the fact that it is possible to target large (compared with historical SNP systems used in forensic genetics [21, 22]) numbers of markers simultaneously from low quantities of DNA [23, 24]. It is also possible to target microhaplotypes with NGS, that is, sets of SNPs located in very close proximity to each other on a chromosome, and these microhaplotype markers have shown promise in forensics for both identification and ancestry purposes [25]. Moreover, the fact that the entire PCR amplicons are analyzed when typing SNP markers with NGS means that any observed variation in the PCR flanking regions will also in effect turn the targeted SNP into a microhaplotype. One example of this is reported by Wendt et al. [25] when studying a Native American population—they found that 22 of the 94 identity SNPs studied contained flanking region variants in the tested population of which 14 were informative (i.e., not in total linkage with one specific allele)—the fact that some identity SNPs are actually microhaplotypes is to be expected and the additional information that this extra variation adds will be valuable for all applications.

### Commercial solutions

The ForenSeq DNA Signature Prep Kit (Illumina, CA) is the first commercially available STR kit for the MiSeq FGx that allows for amplification of up to 153 (DNA primer mix A) or 231 (DNA primer mix B) loci simultaneously. The multiplex assay includes 27 forensic autosomal STRs, 24 Y-STRs, 7-X STRs, the Amelogenin sex marker, and either 94 or 172 SNPs depending on the multiplex formulation. The 172 SNPs can be divided into informative identity, geographical ancestry and phenotypic SNPs. Recent reports describe extensively the performance and technical limitations of this kit using standard quality metrics. The first version of this kit was tested by Churchill et al. in 2015 [26] using the MiSeq desktop sequencer.

The general technical user guidelines were provided by Jager et al. [27], who obtained complete (27 loci) autosomal STR profiles from 89% of the 223 tested standard samples, and from all DNA dilutions between 1 ng and 62.5 pg of input DNA, although their sensitivity study excluded the poorly performing marker D22S1045 and hence a complete profile here consists of 26 autosomal STRs. Sequencing of X- and Y-STRs generated 100% concordance with 1 ng of input material, with only a minor decrease in this figure down to 62.5 pg. Of the 223 standard samples tested, complete SNP genotypes were produced in 87% of samples, with SNP typing demonstrating relative insensitivity to DNA concentration fluctuation within the range of 1 ng–125 pg, with 99.6–99.9% genotype concordance observed. Use of lower DNA amounts resulted in the loss of SNP genotypes, with more than 50% of calls missing if 7.82 pg of gDNA was used. For degraded samples, SNP loci proved more robust than STRs, with the best results being observed when maximal DNA amounts (in max volume of 5 μl) were added rather than the standard recommendation of 1 ng. In the case of DNA mixtures, changes in SNP homo- and heterozygosity were generally detected if the minor contributor was present at 5%, with differences being observed between the SNP subgroups with an increase in heterozygosity of 63% for the identity SNPs and 125% for the ancestry and phenotypic SNPs. Importantly, the authors show that a drop in the number of reads per sample below 85,000 may lead to the increase of ambiguous genotypes and lack of allele calling, which is a valid observation and can be used as a guideline in forensic practice.

Independent validation of the ForenSeq DNA Signature Prep Kit was performed by Xavier et al. (DNA primer Mix A) [28] and Silva et al. (DNA primer Mix B) [29]. Across the two studies, all STR genotypes were concordant with CE results, with the exception of alleles at marker DXS10148 (in line with previous observations [14]) (see Table 1), a marker that has nomenclature issues and has since been removed from the kit. In addition, full STR profiles were obtained for all reference samples across both studies with the exception of two Penta E allele drop-out events in the Mix B set of results. In the sensitivity study with primer Mix A carried out by Xavier et al., the first instance of allele drop-out was detected in marker DX10103 when 250 pg of DNA was used while at 50 pg, a correct profile was obtained for 93.2% of STRs, although drop-out (2.8%), drop-in (0.6%), and discordancy (0.6%) events were observed as well. The poor performance of D22S1045 in this kit has been observed across multiple studies [15, 18, 30–32] and is characterized by a reduction in average read number as the allele size increases, with one suggestion being that the specific ATT repeat motif is the cause of this sequencing issue [30]. Issues with the performance of DYS392 that has a similar ATA repeat motif have also been reported [30, 31]. In the Xavier study, autosomal SNP genotypes were concordant in all triplicates with coverage between 1072 and 20.6 reads per locus, with correct and complete genotyping when more than 100 pg of template was used [33]. Studies overall have highlighted that some autosomal SNPs within this kit perform poorly in specific situations, including rs6955448 [33, 34], rs7041158 [30], and rs2920816 [30] (see Table 1).

**Table 1:** Platform-specific list of frequently reported forensic markers with ambiguous genotypes

Sequencing platforms	Reported problematic markers
MiSeq FGx by Illumina	DYS389II
	DYS448
	DXS10148
	rs459929
	rs1029047
	rs2399332
	rs7251928
	rs7722456
	rs10488710
	T10873C
Ion Torrent	D3S1358
	D7S820
	D8S1179
	rs321198
	rs576261
	rs917118
	rs4530059
	*rs1031825*
MinION by NanoPore	rs733164
	rs873196
	rs1029047
	rs1493232
	*rs1031825*

Common markers are set in italics

Casework-type validation experiments have also been performed with the ForenSeq DNA Signature Prep Kit. Male/female DNA mixtures isolated from buccal swabs were sequenced in triplicates by Xavier et al. [28], and in every case, all markers of the minor contributor were identified, with assessed male contributions of 3.3% (1:20 mixture), 4.4% (1:10 mixture), and 34.8% (1:1 mixture). Silva et al. [29] analyzed 19 mock case samples, prepared using washed and previously worn cotton, nylon, and jeans clothing. One nanogram of DNA from each extract (blood, saliva, or semen) was processed in duplicate using both the ForenSeq DNA Signature Prep protocol and the AmpFISTR Identifiler PCR Amplification Kit. All, except three samples, analyzed on the MiSeq FGx produced complete STR and SNP profiles that were concordant with the donor profile. The genotyping failure in one of these samples was the result of an insufficient read number (39,458 total reads), significantly below the previously suggested threshold of 85,000 per sample. For the other two samples, a blood stain sample with 140,500 reads had a drop-out of one ancestry SNP, and in a washed blood stain sample with 115,311 reads, one identity SNP was missing [34].

ThemoFisher Scientific has designed and released a variety of Precision ID Panels for use on the Ion Torrent platform including those focused on STRs, identity SNPs, ancestry SNPs, and mitochondrial sequencing. The performance of the Ion Torrent instrument suite itself in respect of forensic applications has been tested extensively in the last 4 years for the genotyping of varied selected markers [35–37]. As relating to the currently available commercial panels, extensive validation studies by Pereira et al. [35] and Al-Asfi et al. [36] have been conducted of the 165 SNP Precision ID Ancestry Panel. Both groups investigated the optimal cycling conditions for efficient amplification, finding that 21 cycles was insufficient to produce full 165 SNP profiles at input quantities of less than 1 ng [38] and indeed Pereira et al. found that 30% of loci had a coverage of less than 100 reads even with 1 ng or 500 pg of input DNA [39]. Both studies reported improved results when using 25 PCR cycles, albeit with increased locus imbalance; however, Pereira et al. noted that increased amplification above 25 cycles resulted in unacceptable preferential amplification of shorter DNA fragments. An issue noted in both studies was the uneven read coverage across the 165 SNPs, with rs1296819 specifically featuring in both validations due to the problematically low read count of this marker. Pereira et al. additionally concluded that three SNPs in the assay performed so poorly that they should be excluded from all future work with the panel: rs7722456, rs459929, and rs7251928 (see Table 1). Subsequent studies have validated the associated ancestry prediction software, detailing guidelines to be used in reporting [40], and assessed the performance of the panel with degraded and low-level DNA [38].

The STR and identity SNP panels from ThermoFisher have gone through a number of iterations in both panel design and software [41–46]. The current main STR kit (the Precision ID GlobalFiler NGS STR Panel) comprises all CODIS and European Standard Set loci in addition to nine extra autosomal STR loci (Fig. 1), DYS391, Amelogenin, and the Y-InDel. This kit has been assessed on both the Ion PGM [45] and the Ion S5 [47] instruments. Both studies noted significant imbalance in read coverage between different markers, with Wang et al. [44] suggesting that further modifications to the kit should be undertaken to make coverage levels more consistent across all markers, while Müller et al. [45] demonstrated that marker balance reproducibly changed when the identical panel was run in different laboratories. Observed stutter ratios differed between the studies, with Wang et al. reporting ratios of under 8% for all but three markers, while Müller et al. reporting higher stutter ratios of 10–20% for most markers with 11 loci reaching ratios above 15% (both studies using an input of 1 ng sample DNA)—the reason for this large difference between studies is not immediately obvious, although the use of different instrumentation types (with different associated sequencing reagents) is one possibility. The kit demonstrated good sensitivity in both studies, with most alleles still being recovered with input DNA amounts below 100 pg.

Multiple different groups have carried out validation studies on the Qiagen ID-SNP panel targeting 140 autosomal SNPs using both the MiSeq [24] and Ion Torrent instruments [49, 50]. Of the 140 SNPs, rs1058083 was found to have consistently low coverage across all studies, and in general, the same SNPs performed poorly for all laboratories, indicating the cause to be low amplification efficiency in the initial PCR, although coverage variation for some SNPs (such as rs9951171 which had the 4th lowest coverage for de la Puente et al. [48] but above average coverage for Grandell et al. [22]) could potentially be specific to the sequencing platform used. The analysis of marker rs1029047 was found to be problematic in all studies with both homozygous and heterozygous genotypes showing unexpected allele balance due to the presence of a poly-A tract adjacent to the SNP that caused alignment/sequencing issues, a problem also observed for this SNP when sequenced with an alternative PCR multiplex [44].

All studies observed issues with rs2399332 which manifested as poor heterozygous balance when run on the MiSeq, postulated to be due to a primer binding site mutation [24], and as excess base mis-incorporation in both Ion Torrent studies [49, 50], believed to be due to an adjacent poly-T tract [44] introducing length heteroplasmy during the sequencing process. Various other SNPs manifested with unusual allele balance or high mis-incorporation rates, which appeared to be population or sequencing platform dependent, emphasizing that further work is still needed in order to fully characterize the performance of these markers under variable conditions. One illustration of this is the variability in analysis parameters that were expected or recommended throughout these three different studies, with homozygous genotype thresholds of 95% (i.e., a genotype was called as homozygous when at least 95% of reads were for only 1 allele) [50], 90% [24], or 85% [49].

A variety of offerings produced by Promega, including kits targeting autosomal STRs, Y-STRs, and mitochondrial DNA, have been assessed for use within a forensic setting. The PowerSeq Auto Kit targets 22 commonly used forensic autosomal STRs, one Y-STR, and Amelogenin; this kit is run on the MiSeq platform; however, in contrast with the previously mentioned performance issues with marker D22S1045 in the ForenSeq DNA Signature Prep Kit (Verogen), no problems with D22S1045 have been detailed in any of the PowerSeq validation studies [49, 50], although it should be noted that the total number of markers simultaneously typed with the PowerSeq Auto Kit is 24 compared with over 150 in the ForenSeq Kit. Sensitivity studies with the PowerSeq Auto system demonstrate allele dropout observed only at DNA input amounts of less than 62 pg [51], while optimization experiments for the PowerSeq Auto/Y system have been shown to successfully streamline the laboratory process by removing, modifying, or automating various steps [52].

Across all these validation studies of different commercial genotyping solutions, and different methodology, there is little consensus on universally applicable thresholds for this new technology, and indeed with respect to minimum read coverage requirements, the thresholds can vary considerably. In addition, the development of fully integrated commercial NGS systems for STR typing is currently constrained by the lack of nomenclature guidelines for STR sequence allele variants. Various nomenclature systems have been suggested [11, 53, 54], and the DNA commission of the International Society of Forensic Genetics have published a considerations paper [55], but to date, no firm recommendations have yet been issued. One reason for this delay has been the lack of sequence-based population data for forensic STR markers that can inform this nomenclature discussion; however, this deficiency has been partially addressed in the last few years with the publication of high quality population data [14, 15, 56, 57] and the development of STRseq to catalog STR variants [17].

### mtDNA sequencing

Multiple different PCR strategies exist for amplifying the mitochondrial genome, whether it be in two long segments for whole genome amplification of good-quality DNA [58, 59], multiple smaller fragments (~ 2 kb) for whole genome amplification in partially compromised samples [60], or many overlapping small amplicons for amplification of either the control region [61] or entire genome [62] in degraded samples. NGS analysis with any of these approaches can be carried out using either an enzymatic fragmentation system or by direct sequencing of the PCR products for those methods producing fragments of less than ~ 500 bp. Additionally, commercial solutions are available from ThermoFisher Scientific and Promega that amplify either the control region or the entire mitochondrial genome in 100–400 bp amplicons. The advantages of mitochondrial analysis with MPS include high sensitivity, better heteroplasmy detection [63], and the introduction of a feasible method to sequence the entire mitochondrial genome from forensic quality samples. Detailed analysis of the complete mitochondrial genome significantly increases the discriminatory power of the obtained data compared with control region analysis alone, allowing for maximal resolution of matrilineal geographic ancestry. Additionally, for severely degraded samples, probe capture-NGS methods have been shown to provide results even in the face of very old or extensively damaged mitochondrial DNA [64–66].

The utility of NGS platforms for mtDNA analysis has now been tested in numerous forensic and research labs. In 2014, King et al. [65] described the first attempt to sequence the whole mtDNA genome using Illumina’s MiSeq instrument. In the protocol, mtDNA was processed with the Nextera XT DNA Sample Preparation Kit that allows for fragmentation of the two 8 kb amplicons and addition of adapters via a process called tagmentation (Fig. 2). Although the assay was designed to sequence the genome with the coverage above 5500× per base, the experimental data showed coverage was not equal across the mtGenome and varied within individual mtGenomes. In particular, two regions were characterized by inefficient sequencing: the poly-C stretch in HVII and a portion (< 300 bp around nucleotide 3500) of the NADH dehydrogenase subunit I (ND1) gene. Despite this, replicate analysis showed that reliable variant calls were achieved based on a minimum coverage of only 40 reads. All genotype calls, including point and length heteroplasmy positions, were fully concordant between Sanger sequencing and NGS as tested on nine selected mtDNA samples for HVI/II. The highest polymorphism density was detected in the HVI/HVII regions, as expected, with 25.3% (*n* = 2938) of all the variants called being located within this area. Importantly, King et al. [65] observed that the additional variants present when analyzing the full mitochondrial genome improved the discrimination power and the resolution of mtDNA haplotypes and established the reliability and reproducibility of MPS sequencing in analyzing the mtDNA genome.

Peck et al. [66] performed a comprehensive NGS validation study investigating the reproducibility and concordance of mtDNA sequencing using a MiSeq/Nextera XT workflow. The data of 90 samples were compared with previously performed Sanger sequencing. Both amplicons generated in the MPS approach (approximately 8500 bp long each, DNA input 3 ng–100 pg) were sequenced in duplicate at different laboratories with average read coverage across all bases per sample ranging from a low of 90× to a high of over 1000×, with the coverage distribution consistent across both data sets and haplotypes. A threshold of 10 reads was used for variant calling and only 55 nucleotide positions out of ~ 3 million analyzed in total across both runs were excluded from variant calling due to a coverage of less than this. In line with previous reports, the coverage was reduced around position 3500 and the poly-C stretch of HVII, the latter likely being due to the presence of homopolymeric regions and subsequent difficulties with alignment. Across all identified variants (*n* = 3485) in the first run, only 42 were initially flagged as discordant with previous Sanger sequencing data, which equates to a 99.9996% concordance rate of the assay (across all ~ 1.5 million analyzed position in this run). Of these 42 discordances, only 6 were found to be genuine, representing low-level-point heteroplasmies detected differentially between methods due either to the increase in sensitivity with the MPS analysis or because of normal stochastic variation with such minor variants. Of the 36 false discordances, 32 were found to be due to a low-level DNA mixture in a single sample that had not been detected with Sanger sequencing due to the higher background noise in traditional sequencing electropherograms, while the remaining 4 were due to an alignment artifact. The most problematic genotype calling concerned variant T10873C present in a stretch of five cytosines, which if present at the end of the reads was called a deletion. Similar observations were made in case of other positions if present at the end of reads (e.g., 456, 9477, 9545, 12414, 16362). Due to those shortcomings, the authors strongly suggest optimization of analysis software and alignment algorithms. Additionally, on a practical note, it has been suggested that the software output should indicate the minimum coverage threshold, and the positions that reach this threshold, to avoid reference bases being recorded as present by default for positions failing to reach the minimum coverage.

Recently, McElhoe et al. [61] suggested replacing a standard polymerase used in D-loop amplification with a proofreading polymerase. The obtained results demonstrated that a proofreading polymerase enzyme could be substituted with no associated decrease in amplification efficiency when compared with the standard forensic protocols for mtDNA amplification from buccal swabs and hair shafts, with the advantage that the use of a proofreading polymerase in combination with an enhanced PCR buffer system may be beneficial in the analysis of low-level mtDNA heteroplasmy. A proofreading enzyme should be associated with a reduction in replication error noise, and in this study, point heteroplasmy could then be reproducibly detected above a threshold level of 2%

Similarly, the performance of the Ion Torrent was tested with respect to mtDNA analysis. The first NGS-based attempt of mtDNA genome sequencing was reported in 2012 [68], but it was based on long amplicons, which might not be a suitable approach for the majority of forensic samples. Parson et al. [60] modified the amplification protocol in order to obtain mid-size amplicons 300–500 bp long, utilizing sequencing of 62 amplicons in total to capture the variation in the entire genome. The most versatile mtDNA protocol was proposed by Chaitanya et al. (2015) and implements PCR amplification of 161 DNA fragments, 144–230 bp long [69]. Although more technically challenging than previously proposed tests, it might be the most suitable method for forensic applications dealing with degraded DNA. Validation of this 161 amplicon method with 20 good-quality DNA samples produced average sequencing coverage per base between 1302 and 5637 reads, with an average number of aligned reads per sample of 325,349. The authors applied a 50-read coverage cut-off and using such a threshold achieved full genome coverage for 17/20 samples. The discrepancies between the NGS assay and the known sequence were minimal, although single events were found in nearly each sample, and were listed as: lack of detection of a 9-nt deletion (position 8281–8289), base shifts and insertions in the poly-C and poly-A stretches in the control region and at position 13128, a phantom insertion after base 539 and a false deletion at position 5824. Out of 22 point heteroplasmies, 7 were in agreement with Sanger sequencing, using a 20% threshold. These observations advocate the necessity of manual assessment of heteroplasmy and improvement of bioinformatics analysis in general.

Using Ion Torrent, Ma et al. confirmed that mtGenome sequencing can increase the discrimination potential of mtDNA, simultaneously corroborating the difficulty with sequencing of poly-C tracts (35552–3575; 8605–8625).

## Conclusions and future perspectives

The ability of genotyping different DNA markers simultaneously using NGS benchtop sequencers has been demonstrated by The ForenSeq DNA Signature Prep Kit which combines synchronized typing of STR and SNP markers. But one of the most forensically attractive features of NGS-based technology is the possibility of simultaneous analysis of different types of nucleic acids. In the last decade, it has been shown that analysis of RNA is also of forensic relevance with multiple applications such as tissue and body fluid identification, determination of time of death, etc. (see Fig. 2). Although RNA is more difficult to work with, mainly due to its susceptibility to degradation by ubiquitously present RNAses, its usefulness gains more significance and becomes more visible specifically due to recent technological advances.

Current technological and bioinformatics developments allow to correlate transcriptomic data with DNA sequencing and methylation patterns, which aids the extraction of maximal biological/phenotypic information from the same sample. Moreover, integrative analysis of RNA and DNA sequencing provides additional verification of variant calls in the coding regions. Traditionally, due to the limited amount of starting material simultaneous analysis of multiple nucleic acids has not been utilized in forensic settings. If sample concentration was sufficient, the standard isolation protocols did not include synchronous but consecutive RNA and DNA extraction or alternatively proposed dividing the samples for the separate isolations [70, 71].

It has been shown that limited concentration of nucleic acids can influence the quality of the library preparation and sequencing; hence, sample splitting is not always the preferred choice [70–73]. Also, recent reports describe a number of protocol improvements in case of non-human sample testing [70–72].

In 2015, Zubakov et al. for the first time attempted to sequence DNA and RNA markers simultaneously using Ion Torrent [74]. Although the performed experiments were crude and require further fine tuning, importantly, the authors show that such analysis is possible. The goal was to identify 9 autosomal STRs and 12 RNA markers enabling identification and discrimination of body fluids. The DNA analysis was performed according to standard protocol with 1 ng of template, while 10 ng of RNA was first transcribed to cDNA, and 150 pb long amplicons were subjected to regular library preparation protocol. The amplicons allowed for gene expression analysis of the following mRNAs: two housekeeping genes (*HPRT1*, *SDHA*), markers of menstrual blood (*MMP10*, *MMP11*), peripheral blood (*ALAS2*, *SPTB*), saliva (*HTN3*, *STATH*), semen (*PMR1*, *TGM4*), skin (*CCL27*, *LCE1C*), and vaginal secretion (*CYP2B7P1*, *MUC4*). Surprisingly, in the runs, only half of the obtained reads met the standard quality requirements and were usable for the downstream analysis. The authors suggest it might be due to a low number of samples used in relation to the sequencing capacity of the chip in combination with the barcoding implemented in the experiments. The specificity of the mRNA markers was very high, and although a few markers were expressed in multiple samples (e.g., CYP2B7P1, vaginal mucosa/menstrual blood), the combination of the expression data allowed for unambiguous tissue identification as demonstrated by binary logistic expression analysis. The idea of multiple nucleic acid isolation appeals to many forensic laboratories, and there are reports that aim to optimize their simultaneous isolation, exemplified by Day et al. [82].

Recently published NGS protocols enable us to utilize low-input samples for simultaneous analysis of RNA and DNA (see Table 2). Although the procedures are relatively more intricate and potentially time consuming, these new options might gain a significant relevance in forensics. While Simul-seq allows for generation of good-quality library preparations and sequencing from 50 ng DNA and 100 ng RNA and Gel-seq from 100 to 1000 cells, the remaining assays: GT-seq, DR-seq, and SIDR-seq were implemented in single-cell DNA/RNA sequencing. While simultaneous testing of forensic samples for the remaining types of nucleic acids might not be visible when the amount of the collected material is limited, recent reports suggest different RNA types could be suitable as forensic markers [76]. Small RNAs are short, 1821 nt long molecules that are conserved throughout the species. Although small RNA profiling might not be informative as stand-alone analysis, it could provide forensically relevant information, following the determination of sample origin. The first qPCR-based body fluid analysis implementing small RNA profiling was proposed by Hanson et al., but the findings were difficult to reproduce in other forensic laboratories [77]. In 2018, Tian et al. validated a small RNA expression test to distinguish semen and non-semen body fluids; moreover, the analysis of the six molecules: miR-10a, miR-10b, miR-135a, miR-135b, miR-888, and miR891a, allowed for discrimination between normal and infertile semen [76]. While the proposed assays target only candidate small RNA, the complete profiles, derived from any tissue or fluid, might provide additional information not only on the tissue/fluid type but also on the health status of the individual, since small RNA expression is altered in many diseases, including cancer [78]. Similar observations have been made for many different RNA subtypes, including long non-coding RNAs [78].

**Table 2:** Comparison of recently developed NGS-based assays’ potential applicability to forensics

	DR-Seq	G&T Seq	SIDR	Simuel-Seq	Gel-Seq
Input	Single cell	Single cell	Single cell	50–100 ng	100–1000 cells
DNA/RNA separation	Specific barcoding in first PCR	PolyA tail separation	Antibgody = based in combination with conjugated bead surface	Differential adapters	Size-based separation
DNA/RNA amplification	1 tube PCR	Separate amplification of (A) RNA and DNA amplification—method of choice	Repli-G; single cell (Qiagen)	PCR	Separate qPCR reactions
Library input	10 ng	1–5 ng	1–5 ng	1–500 ng	0.2 ng
Library preparation	NEBNext Ultra DNA Library Prep Kit (Illumina)	Nextera XT DNA Sample Prep Kit (Illumina)	Nextera XT DNA Sample Prep Kit (Illumina)	Nextera DNA Prep Kit (Illumina)	Hoople et al. [83]
	Poly A primer				Gel preparation
Potential drawbacks	No isolation of remaining RNA subpopulation	Poly A separation	RNA separation and elution	rRNA depletion	Non-commercial library prep protocol
Reference	Day et al. [82]	Macaulay et al. (2015)	Han et al. [79]	Reuter et al. [78]	Hoople et al. [83]

Though sequencing by synthesis technology (SBS) dominates the NGS market at the moment, there are many attempts to introduce different approaches to sequencing and to overcome the shortcomings of the 2nd-generation sequencing. Currently, the most promising technology is nanopore sequencing based on the detection of ionic current change while a nucleotide passes through a pore. This technology eliminates PCR amplification (which may lead to preferential DNA amplification) and the cyclic mode of sequencing is replaced by sequencing in real time with reads up to 10,000 bp. Although still inferior to commonly used MPS sequencing (error rates, costs, time of data analysis, allelic imbalance), the technology is very promising, and if optimized, may be utilized in many research fields including forensics. Recently, a portable nanopore-based sequencer MinION (Oxford Nanopore Technologies) has been introduced to the market and Cornelis et al. as first tested its utility in forensic SNP testing [79]. The authors set out to analyze a single female sample for 52 SNPs developed by the SNPforID consortium, and the data was compared with the genotypes obtained by Illumina sequencing; 2.5 ng of template DNA was used for generation of amplicons, which were further pooled and randomly concatenated in order to create longer DNA fragments. After end-repair, leader and hairpin adaptors were added and tailed with poly-A. The data was retrieved as fast five files and analyzed with Metrichor service, which uses Recurrent Neural Network for base calling. The average number of reads per locus was 29,888, with average coverage of 17,933 (SD = 8452), which was sufficient for SNP calling. On average, 60% of the obtained reads were mapped to the reference sequences. For rs1029047, a decreased number of reads was obtained, with only 12% of mapped reads, likely due to the position in the poly-A stretch. The difficulties in SNP sequencing and mapping of homopolymeric regions were also reported by Loman et al. [71]. Two loci with SNPs between or inside polymer stretches, rs143232 and rs1031825 (see Table 1), displayed significant allelic imbalance, a result also previously reported by Ion Torrent users [71]. Only one of the two markers rs1031825 was incorrectly called (heterozygote), as compared with Illumina genotyping (homozygote). Although only SNP genotyping was tested using the MinION, the significance of this report is twofold: (1) it demonstrates that it is possible to correctly call the forensic SNPs using a nanopore sequencer if homopolymer stretches are avoided and (2) a portable, low-throughput sequencing device such as the MinION might be of particular convenience in forensic analyses. A recent proof-of-concept study [69] testing the applicability of this technology to DNA identification established that a match of 99.9% accuracy could be achieved in 3 min if comparing a novel sample against a database of previously sequenced samples. While the approach used in this study is incompatible with current forensic methodologies and databases, it demonstrates the potential for this form of rapid, field-based identification in both military and law enforcement situations in time-critical cases and where access to standard DNA profiling technology is limited.
